# (*meso*-5,5,7,12,12,14-Hexamethyl-1,4,8,11-tetra­aza­cyclo­tetra­deca­ne)nickel(II) bis­[*O*,*O*′-bis­(4-methyl­phen­yl) thio­phosphate]

**DOI:** 10.1107/S1600536810051184

**Published:** 2010-12-11

**Authors:** Yang-Guang Xiang, Bin Xie, Li-Ke Zou, Jian-Shen Feng, Chuan Lai

**Affiliations:** a College of Chemistry and Pharmaceutical Engineering, Sichuan University of Science and Engineering, 643000 Zigong, Sichuan, People’s Republic of China; b Research Institute of Functional Material, Sichuan University of Science and Engineering, 643000 Zigong, Sichuan, People’s Republic of China

## Abstract

In the centrosymmetric title complex, [Ni(C_16_H_36_N_4_)](C_14_H_14_O_3_PS)_2_, the Ni^II^ ion is coordinated by four N atoms and two O atoms within a slightly distorted NiN_4_O_2_ octa­hedral geometry. The asymmetric unit consits of one Ni^II^ ion that is located on a center of inversion, half of the macrocylic ligand and one anion occupying general positions. Intra­molecular N—H⋯O and N—H⋯S hydrogen bonding is found between the macrocyclic ligand and the monothio­phosphate anion.

## Related literature

For the synthesis of *O*,*O*′-bis­(4-methyl­phen­yl) monothio­phos­phate, see: Pesin & Khaletakii (1961 ▶). For related structures, see: Feng *et al.* (2010 ▶); He *et al.* (2010 ▶); Zou *et al.* (2010 ▶).
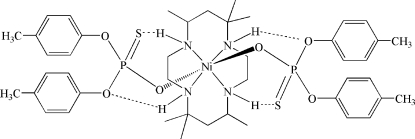


## Experimental

### 

#### Crystal data

[Ni(C_16_H_36_N_4_)](C_14_H_14_O_3_PS)_2_
*M* *_r_* = 929.76
Monoclinic, 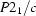

*a* = 10.977 (2) Å
*b* = 16.360 (3) Å
*c* = 12.767 (3) Å
β = 94.85 (3)°
*V* = 2284.6 (8) Å^3^
*Z* = 2
Mo *K*α radiation
μ = 0.64 mm^−1^
*T* = 113 K
0.24 × 0.23 × 0.22 mm

#### Data collection

Rigaku Saturn CCD area-detector diffractometer
Absorption correction: multi-scan (*CrystalClear*; Rigaku, 2005 ▶) *T*_min_ = 0.862, *T*_max_ = 0.873
18654 measured reflections
5376 independent reflections
2665 reflections with *I* > 2σ(*I*)
*R*_int_ = 0.101

#### Refinement

*R*[*F* ^2^ > 2σ(*F* ^2^)] = 0.053
*wR*(*F* ^2^) = 0.122
*S* = 0.99
5376 reflections
281 parameters
H atoms treated by a mixture of independent and constrained refinement
Δρ_max_ = 0.97 e Å^−3^
Δρ_min_ = −0.91 e Å^−3^

### 

Data collection: *CrystalClear* (Rigaku, 2005 ▶); cell refinement: *CrystalClear*; data reduction: *CrystalClear*; program(s) used to solve structure: *SHELXS97* (Sheldrick, 2008 ▶); program(s) used to refine structure: *SHELXL97* (Sheldrick, 2008 ▶); molecular graphics: *ORTEP-3 for Windows* (Farrugia, 1997 ▶); software used to prepare material for publication: *SHELXL97*.

## Supplementary Material

Crystal structure: contains datablocks I, global. DOI: 10.1107/S1600536810051184/nc2207sup1.cif

Structure factors: contains datablocks I. DOI: 10.1107/S1600536810051184/nc2207Isup2.hkl

Additional supplementary materials:  crystallographic information; 3D view; checkCIF report

## Figures and Tables

**Table 1:** Hydrogen-bond geometry (Å, °)

*D*—H⋯*A*	*D*—H	H⋯*A*	*D*⋯*A*	*D*—H⋯*A*
N1—H1⋯S1^i^	0.92 (3)	2.66 (3)	3.574 (3)	171 (3)
N2—H2⋯O2	0.96 (3)	2.27 (3)	3.234 (3)	178 (3)

Symmetry code: (i) 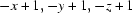.

**Fig. 1.**
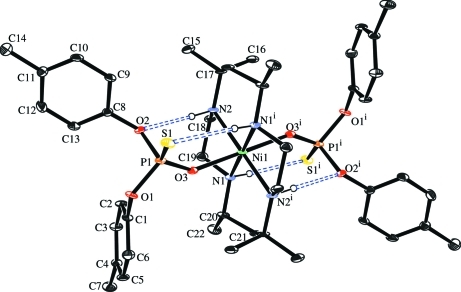
The molecular structure of the title complex, showing the atom-numbering scheme with displacement ellipsoids drawn at 30% probability level. H atoms on N are represented as small spheres of arbitary radii and H atoms on C have been omitted for the sake of clarity. Hydrogen-bonds are shown as dashed lines. [Symmetry code: (i) -x + 1, -y + 1, -z + 1].

## supplementary crystallographic information

### Comment

In our research on tetramine macrocycles transition metal complexes as mimetic hydrolases, we have recently reported several structures of their adducts with *O*,*O*'-dialkyldithiophosphate (He *et al.*, 2010; Feng *et al.*, 2010; Zou *et al.*, 2010). Herein, we report the structure of an analogous *O*,*O*'-dialkylmonothiophosphate adducts, [Ni(*meso*-hmta)][OP(S)(OC_6_H_4_Me-4)_2_]_2_, where *meso*-hmta is *meso*-5,5,7,12,12,14-Hexamethyl-1,4,8,11-tetraazacyclotetradecane and (4-MeC_6_H_4_O)_2_(S)PO^-^ is *O*,*O*'-bis(4-methylphenyl) monothiophosphate.

In the crystal structure of the title complex, the Ni^II^ ion is located on a center of inversion and possesses a slightly distorted NiN_4_O_2_ octahedral geometry (Fig. 1). The tetraamine macrocycle *meso*-hmta folds around the Ni^II^ centre at equatorial position and two O atoms from symmetry related *O*,*O*'-bis(4-methylphenyl) monothiophosphates are located in axial positions (Fig. 1). Intramolecular N—H···O and N—H···S hydrogen bonds are present between *meso*-hmta and monothiophosphates ligands (table 1). Furthermore, there exists a pair of symmetry related weak intermolecular C—H···π interactions for (C14—H14C)···C3(phenyl) between each couple of adjacent monothiophosphate ligands, which link the molecules into one-dimensional chains along [010]. The P1—O3 and P1—S1 bond lengths are 1.491 (2) and 1.9372 (14) Å respectively, corresponding to a delocalization of the negative charge over the O3—P1—S1 fragment.

### Experimental

The ammonium *O*,*O*'-bis(4-methylphenyl)monothiophosphate was prepared according to the procedure described by Pesin (1961).

A solution of *meso*-5,5,7,12,12,14- hexamethyl-1,4,8,11- tetraazacyclotetradecane dihydrate (0.64 g, 2 mmol) and Ni(OAc)_2_.4H_2_O (0.50 g, 2 mmol) in 20 mL methanol was added to a solution of ammonium *O*,*O*'-bis(4-methylphenyl)monothiophosphate (4 mmol, 1.24 g) in 60 mL methanol. The mixture was refluxed for 6 h at 353 K and then filtered after cooling to room temperature. The filtrate was kept at room temperature and orange block crystals were obtained after 4 weeks.

### Refinement

H atoms attached to C atoms were fixed geometrically and treated as riding, with C—H = 1.00Å (methine), 0.99Å (methylene), 0.98Å (methyl), 0.95Å (aromatic). The *U*_iso_(H) = 1.5*U*_eq_(C) for methyl groups and *U*_iso_(H) = 1.2*U*_eq_(C) for all other carbon bound H atoms. H atoms on N atoms were located in the difference map and refined isotropically.

### Crystal data

**Table:**

[Ni(C_16_H_36_N_4_)](C_14_H_14_O_3_PS)_2_	*F*(000) = 988
*M**_r_* = 929.76	*D*_x_ = 1.352 Mg m^−^^3^
Monoclinic, *P*2_1_/*c*	Mo *K*α radiation, λ = 0.71073 Å
Hall symbol: -P 2ybc	Cell parameters from 7232 reflections
*a* = 10.977 (2) Å	θ = 2.2–27.9°
*b* = 16.360 (3) Å	µ = 0.64 mm^−^^1^
*c* = 12.767 (3) Å	*T* = 113 K
β = 94.85 (3)°	Block, orange
*V* = 2284.6 (8) Å^3^	0.24 × 0.23 × 0.22 mm
*Z* = 2	

### Data collection

**Table:**

Rigaku Saturn CCD area-detector diffractometer	5376 independent reflections
Radiation source: rotating anode	2665 reflections with *I* > 2σ(*I*)
confocal	*R*_int_ = 0.101
Detector resolution: 7.31 pixels mm^-1^	θ_max_ = 27.9°, θ_min_ = 2.2°
φ and ω scans	*h* = −14→13
Absorption correction: multi-scan (*CrystalClear*; Rigaku, 2005)	*k* = −21→19
*T*_min_ = 0.862, *T*_max_ = 0.873	*l* = −16→16
18654 measured reflections	

### Refinement

**Table:**

Refinement on *F*^2^	Primary atom site location: structure-invariant direct methods
Least-squares matrix: full	Secondary atom site location: difference Fourier map
*R*[*F*^2^ > 2σ(*F*^2^)] = 0.053	Hydrogen site location: mixed
*wR*(*F*^2^) = 0.122	H atoms treated by a mixture of independent and constrained refinement
*S* = 0.99	*w* = 1/[σ^2^(*F*_o_^2^) + (0.0244*P*)^2^] where *P* = (*F*_o_^2^ + 2*F*_c_^2^)/3
5376 reflections	(Δ/σ)_max_ < 0.001
281 parameters	Δρ_max_ = 0.97 e Å^−^^3^
0 restraints	Δρ_min_ = −0.91 e Å^−^^3^

### Special details

**Table:**

Geometry. All esds (except the esd in the dihedral angle between two l.s. planes) are estimated using the full covariance matrix. The cell esds are taken into account individually in the estimation of esds in distances, angles and torsion angles; correlations between esds in cell parameters are only used when they are defined by crystal symmetry. An approximate (isotropic) treatment of cell esds is used for estimating esds involving l.s. planes.
Refinement. Refinement of F^2^ against ALL reflections. The weighted R-factor wR and goodness of fit S are based on F^2^, conventional R-factors R are based on F, with F set to zero for negative F^2^. The threshold expression of F^2^ > 2sigma(F^2^) is used only for calculating R-factors(gt) etc. and is not relevant to the choice of reflections for refinement. R-factors based on F^2^ are statistically about twice as large as those based on F, and R- factors based on ALL data will be even larger.

### Fractional atomic coordinates and isotropic or equivalent isotropic displacement parameters (Å^2^)

**Table:**

	*x*	*y*	*z*	*U*_iso_*/*U*_eq_	
Ni1	0.5000	0.5000	0.5000	0.02073 (17)	
P1	0.49238 (7)	0.69655 (5)	0.59137 (7)	0.0216 (2)	
S1	0.57082 (7)	0.69510 (5)	0.73310 (7)	0.0288 (2)	
O1	0.38094 (17)	0.76174 (13)	0.58832 (18)	0.0236 (6)	
O2	0.58179 (16)	0.73444 (12)	0.50834 (17)	0.0214 (5)	
O3	0.44815 (16)	0.61948 (12)	0.53863 (17)	0.0223 (5)	
N1	0.3899 (2)	0.50845 (17)	0.3600 (2)	0.0219 (6)	
H1	0.395 (3)	0.4578 (19)	0.329 (3)	0.035 (10)*	
N2	0.6334 (2)	0.55494 (16)	0.4156 (2)	0.0215 (7)	
H2	0.616 (3)	0.6087 (18)	0.441 (3)	0.030 (9)*	
C1	0.2889 (3)	0.76449 (19)	0.5047 (3)	0.0223 (8)	
C2	0.3144 (3)	0.76956 (19)	0.4017 (3)	0.0246 (8)	
H2A	0.3966	0.7682	0.3837	0.030*	
C3	0.2189 (3)	0.7767 (2)	0.3242 (3)	0.0275 (8)	
H3	0.2366	0.7805	0.2528	0.033*	
C4	0.0970 (3)	0.77851 (19)	0.3485 (3)	0.0250 (8)	
C5	0.0746 (3)	0.7723 (2)	0.4531 (3)	0.0278 (9)	
H5	−0.0073	0.7727	0.4719	0.033*	
C6	0.1699 (3)	0.7656 (2)	0.5310 (3)	0.0296 (9)	
H6	0.1531	0.7617	0.6026	0.035*	
C7	−0.0083 (3)	0.7901 (2)	0.2647 (3)	0.0389 (10)	
H7A	−0.0184	0.7405	0.2218	0.058*	
H7C	0.0093	0.8365	0.2198	0.058*	
H7B	−0.0837	0.8009	0.2984	0.058*	
C8	0.6458 (3)	0.80740 (19)	0.5269 (3)	0.0217 (8)	
C9	0.7636 (3)	0.8040 (2)	0.5759 (3)	0.0267 (8)	
H9	0.7978	0.7536	0.6008	0.032*	
C10	0.8303 (3)	0.8763 (2)	0.5877 (3)	0.0307 (9)	
H10	0.9115	0.8743	0.6199	0.037*	
C11	0.7829 (3)	0.9500 (2)	0.5545 (3)	0.0295 (9)	
C12	0.6644 (3)	0.9517 (2)	0.5073 (3)	0.0341 (9)	
H12	0.6292	1.0023	0.4843	0.041*	
C13	0.5966 (3)	0.8805 (2)	0.4934 (3)	0.0301 (9)	
H13	0.5157	0.8826	0.4605	0.036*	
C14	0.8567 (3)	1.0273 (2)	0.5677 (3)	0.0458 (11)	
H14A	0.8793	1.0458	0.4989	0.069*	
H14B	0.9310	1.0169	0.6139	0.069*	
H14C	0.8080	1.0697	0.5989	0.069*	
C15	0.8417 (3)	0.6094 (2)	0.3973 (3)	0.0361 (10)	
H15A	0.8263	0.6104	0.3206	0.054*	
H15C	0.9291	0.6010	0.4164	0.054*	
H15B	0.8161	0.6615	0.4264	0.054*	
C16	0.8058 (3)	0.45790 (19)	0.3946 (3)	0.0322 (9)	
H16A	0.7479	0.4153	0.4115	0.048*	
H16C	0.8882	0.4429	0.4239	0.048*	
H16B	0.8050	0.4635	0.3182	0.048*	
C17	0.7688 (3)	0.5393 (2)	0.4421 (3)	0.0264 (8)	
C18	0.5894 (3)	0.5475 (2)	0.3036 (3)	0.0292 (9)	
H18A	0.6039	0.4914	0.2783	0.035*	
H18B	0.6335	0.5864	0.2610	0.035*	
C19	0.4532 (3)	0.5665 (2)	0.2936 (3)	0.0286 (9)	
H19B	0.4395	0.6233	0.3167	0.034*	
H19A	0.4202	0.5612	0.2193	0.034*	
C20	0.2599 (3)	0.5272 (2)	0.3719 (3)	0.0271 (8)	
H20	0.2554	0.5804	0.4102	0.033*	
C21	0.2064 (3)	0.4599 (2)	0.4382 (3)	0.0284 (9)	
H21B	0.1166	0.4606	0.4221	0.034*	
H21A	0.2360	0.4069	0.4129	0.034*	
C22	0.1847 (3)	0.5349 (2)	0.2649 (3)	0.0393 (10)	
H22C	0.2188	0.5788	0.2240	0.059*	
H22B	0.0996	0.5477	0.2762	0.059*	
H22A	0.1880	0.4832	0.2266	0.059*	

### Atomic displacement parameters (Å^2^)

**Table:**

	*U*^11^	*U*^22^	*U*^33^	*U*^12^	*U*^13^	*U*^23^
Ni1	0.0054 (3)	0.0286 (3)	0.0277 (4)	−0.0020 (2)	−0.0018 (2)	0.0003 (3)
P1	0.0101 (4)	0.0288 (5)	0.0254 (6)	0.0011 (4)	−0.0012 (4)	0.0002 (4)
S1	0.0183 (5)	0.0418 (6)	0.0253 (6)	0.0041 (4)	−0.0039 (4)	0.0001 (4)
O1	0.0097 (11)	0.0319 (13)	0.0286 (16)	0.0078 (10)	−0.0013 (10)	−0.0016 (10)
O2	0.0102 (11)	0.0251 (12)	0.0287 (15)	−0.0035 (9)	−0.0003 (10)	0.0007 (10)
O3	0.0090 (11)	0.0259 (12)	0.0311 (16)	−0.0025 (9)	−0.0037 (10)	−0.0022 (10)
N1	0.0093 (14)	0.0271 (16)	0.0282 (19)	−0.0002 (12)	−0.0046 (12)	0.0010 (13)
N2	0.0068 (13)	0.0291 (17)	0.0287 (19)	−0.0005 (12)	0.0022 (12)	0.0010 (13)
C1	0.0087 (16)	0.0267 (18)	0.030 (2)	0.0015 (14)	−0.0059 (15)	−0.0003 (15)
C2	0.0074 (16)	0.037 (2)	0.028 (2)	−0.0006 (15)	−0.0028 (15)	−0.0053 (16)
C3	0.0164 (18)	0.041 (2)	0.025 (2)	0.0003 (16)	0.0000 (16)	−0.0053 (16)
C4	0.0109 (17)	0.0274 (19)	0.035 (2)	−0.0008 (14)	−0.0083 (15)	−0.0062 (15)
C5	0.0088 (17)	0.037 (2)	0.037 (3)	−0.0005 (15)	0.0000 (16)	0.0045 (17)
C6	0.0208 (19)	0.037 (2)	0.031 (2)	0.0005 (16)	0.0033 (17)	0.0057 (17)
C7	0.0173 (19)	0.053 (3)	0.044 (3)	0.0011 (18)	−0.0110 (18)	−0.005 (2)
C8	0.0154 (17)	0.0243 (18)	0.025 (2)	−0.0019 (14)	−0.0005 (15)	−0.0022 (14)
C9	0.0120 (17)	0.0276 (19)	0.039 (3)	0.0033 (14)	−0.0042 (15)	−0.0014 (16)
C10	0.0104 (17)	0.035 (2)	0.045 (3)	0.0011 (15)	−0.0036 (16)	−0.0060 (18)
C11	0.0216 (19)	0.0244 (19)	0.042 (3)	−0.0039 (15)	0.0015 (17)	−0.0068 (17)
C12	0.028 (2)	0.0241 (19)	0.049 (3)	0.0052 (16)	−0.0078 (18)	0.0010 (17)
C13	0.0134 (17)	0.034 (2)	0.041 (3)	0.0020 (16)	−0.0046 (16)	0.0004 (17)
C14	0.033 (2)	0.032 (2)	0.071 (4)	−0.0046 (18)	0.002 (2)	−0.007 (2)
C15	0.0163 (18)	0.040 (2)	0.052 (3)	−0.0080 (17)	0.0056 (18)	−0.0021 (19)
C16	0.0117 (17)	0.039 (2)	0.046 (3)	−0.0004 (16)	0.0041 (16)	−0.0026 (18)
C17	0.0038 (15)	0.032 (2)	0.043 (3)	−0.0026 (14)	0.0005 (15)	−0.0032 (17)
C18	0.0139 (17)	0.037 (2)	0.038 (3)	−0.0031 (15)	0.0064 (16)	0.0042 (17)
C19	0.0194 (18)	0.035 (2)	0.030 (2)	0.0013 (16)	−0.0020 (16)	0.0037 (16)
C20	0.0071 (16)	0.038 (2)	0.036 (2)	0.0036 (15)	−0.0035 (15)	0.0000 (17)
C21	0.0051 (16)	0.037 (2)	0.043 (3)	−0.0018 (15)	−0.0024 (15)	−0.0024 (17)
C22	0.0195 (19)	0.051 (3)	0.045 (3)	0.0058 (18)	−0.0129 (17)	0.004 (2)

### Geometric parameters (Å, °)

**Table:**

Ni1—N1	2.076 (3)	C9—H9	0.9500
Ni1—N1^i^	2.076 (3)	C10—C11	1.367 (4)
Ni1—N2	2.093 (2)	C10—H10	0.9500
Ni1—N2^i^	2.093 (2)	C11—C12	1.388 (4)
Ni1—O3^i^	2.106 (2)	C11—C14	1.504 (4)
Ni1—O3	2.106 (2)	C12—C13	1.385 (4)
P1—O3	1.491 (2)	C12—H12	0.9500
P1—O1	1.621 (2)	C13—H13	0.9500
P1—O2	1.627 (2)	C14—H14A	0.9800
P1—S1	1.9372 (14)	C14—H14B	0.9800
O1—C1	1.407 (4)	C14—H14C	0.9800
O2—C8	1.395 (3)	C15—C17	1.535 (4)
N1—C20	1.480 (4)	C15—H15A	0.9800
N1—C19	1.485 (4)	C15—H15C	0.9800
N1—H1	0.92 (3)	C15—H15B	0.9800
N2—C18	1.474 (4)	C16—C17	1.533 (4)
N2—C17	1.518 (4)	C16—H16A	0.9800
N2—H2	0.96 (3)	C16—H16C	0.9800
C1—C2	1.370 (4)	C16—H16B	0.9800
C1—C6	1.376 (4)	C17—C21^i^	1.529 (5)
C2—C3	1.384 (4)	C18—C19	1.522 (4)
C2—H2A	0.9500	C18—H18A	0.9900
C3—C4	1.399 (4)	C18—H18B	0.9900
C3—H3	0.9500	C19—H19B	0.9900
C4—C5	1.382 (5)	C19—H19A	0.9900
C4—C7	1.519 (4)	C20—C21	1.536 (4)
C5—C6	1.385 (4)	C20—C22	1.540 (4)
C5—H5	0.9500	C20—H20	1.0000
C6—H6	0.9500	C21—C17^i^	1.529 (5)
C7—H7A	0.9800	C21—H21B	0.9900
C7—H7C	0.9800	C21—H21A	0.9900
C7—H7B	0.9800	C22—H22C	0.9800
C8—C13	1.366 (4)	C22—H22B	0.9800
C8—C9	1.390 (4)	C22—H22A	0.9800
C9—C10	1.392 (4)		
			
N1—Ni1—N1^i^	180.0	C11—C10—C9	122.2 (3)
N1—Ni1—N2	84.83 (11)	C11—C10—H10	118.9
N1^i^—Ni1—N2	95.17 (11)	C9—C10—H10	118.9
N1—Ni1—N2^i^	95.17 (11)	C10—C11—C12	118.1 (3)
N1^i^—Ni1—N2^i^	84.83 (11)	C10—C11—C14	121.4 (3)
N2—Ni1—N2^i^	180.0	C12—C11—C14	120.6 (3)
N1—Ni1—O3^i^	90.54 (10)	C13—C12—C11	120.8 (3)
N1^i^—Ni1—O3^i^	89.46 (10)	C13—C12—H12	119.6
N2—Ni1—O3^i^	93.63 (9)	C11—C12—H12	119.6
N2^i^—Ni1—O3^i^	86.37 (9)	C8—C13—C12	120.2 (3)
N1—Ni1—O3	89.46 (10)	C8—C13—H13	119.9
N1^i^—Ni1—O3	90.54 (10)	C12—C13—H13	119.9
N2—Ni1—O3	86.37 (9)	C11—C14—H14A	109.5
N2^i^—Ni1—O3	93.63 (9)	C11—C14—H14B	109.5
O3^i^—Ni1—O3	179.999 (1)	H14A—C14—H14B	109.5
O3—P1—O1	109.22 (12)	C11—C14—H14C	109.5
O3—P1—O2	102.67 (12)	H14A—C14—H14C	109.5
O1—P1—O2	103.25 (11)	H14B—C14—H14C	109.5
O3—P1—S1	120.90 (10)	C17—C15—H15A	109.5
O1—P1—S1	107.79 (10)	C17—C15—H15C	109.5
O2—P1—S1	111.61 (9)	H15A—C15—H15C	109.5
C1—O1—P1	122.1 (2)	C17—C15—H15B	109.5
C8—O2—P1	122.6 (2)	H15A—C15—H15B	109.5
P1—O3—Ni1	143.74 (12)	H15C—C15—H15B	109.5
C20—N1—C19	115.3 (2)	C17—C16—H16A	109.5
C20—N1—Ni1	115.0 (2)	C17—C16—H16C	109.5
C19—N1—Ni1	105.51 (19)	H16A—C16—H16C	109.5
C20—N1—H1	108.7 (19)	C17—C16—H16B	109.5
C19—N1—H1	106 (2)	H16A—C16—H16B	109.5
Ni1—N1—H1	105 (2)	H16C—C16—H16B	109.5
C18—N2—C17	115.9 (2)	N2—C17—C21^i^	108.1 (2)
C18—N2—Ni1	106.15 (18)	N2—C17—C16	110.2 (3)
C17—N2—Ni1	121.8 (2)	C21^i^—C17—C16	111.8 (3)
C18—N2—H2	110 (2)	N2—C17—C15	108.8 (3)
C17—N2—H2	107.0 (18)	C21^i^—C17—C15	108.2 (3)
Ni1—N2—H2	93.0 (17)	C16—C17—C15	109.7 (3)
C2—C1—C6	120.6 (3)	N2—C18—C19	107.6 (3)
C2—C1—O1	122.5 (3)	N2—C18—H18A	110.2
C6—C1—O1	116.8 (3)	C19—C18—H18A	110.2
C1—C2—C3	119.2 (3)	N2—C18—H18B	110.2
C1—C2—H2A	120.4	C19—C18—H18B	110.2
C3—C2—H2A	120.4	H18A—C18—H18B	108.5
C2—C3—C4	121.6 (3)	N1—C19—C18	109.0 (3)
C2—C3—H3	119.2	N1—C19—H19B	109.9
C4—C3—H3	119.2	C18—C19—H19B	109.9
C5—C4—C3	117.6 (3)	N1—C19—H19A	109.9
C5—C4—C7	120.2 (3)	C18—C19—H19A	109.9
C3—C4—C7	122.1 (3)	H19B—C19—H19A	108.3
C4—C5—C6	121.0 (3)	N1—C20—C21	108.9 (3)
C4—C5—H5	119.5	N1—C20—C22	112.0 (3)
C6—C5—H5	119.5	C21—C20—C22	110.3 (3)
C1—C6—C5	120.0 (3)	N1—C20—H20	108.5
C1—C6—H6	120.0	C21—C20—H20	108.5
C5—C6—H6	120.0	C22—C20—H20	108.5
C4—C7—H7A	109.5	C17^i^—C21—C20	120.1 (3)
C4—C7—H7C	109.5	C17^i^—C21—H21B	107.3
H7A—C7—H7C	109.5	C20—C21—H21B	107.3
C4—C7—H7B	109.5	C17^i^—C21—H21A	107.3
H7A—C7—H7B	109.5	C20—C21—H21A	107.3
H7C—C7—H7B	109.5	H21B—C21—H21A	106.9
C13—C8—C9	120.3 (3)	C20—C22—H22C	109.5
C13—C8—O2	121.1 (3)	C20—C22—H22B	109.5
C9—C8—O2	118.6 (3)	H22C—C22—H22B	109.5
C8—C9—C10	118.4 (3)	C20—C22—H22A	109.5
C8—C9—H9	120.8	H22C—C22—H22A	109.5
C10—C9—H9	120.8	H22B—C22—H22A	109.5
			
O3—P1—O1—C1	−32.3 (3)	C2—C3—C4—C7	177.2 (3)
O2—P1—O1—C1	76.4 (2)	C3—C4—C5—C6	0.8 (5)
S1—P1—O1—C1	−165.4 (2)	C7—C4—C5—C6	−176.9 (3)
O3—P1—O2—C8	−179.0 (2)	C2—C1—C6—C5	−0.4 (5)
O1—P1—O2—C8	67.5 (2)	O1—C1—C6—C5	176.8 (3)
S1—P1—O2—C8	−48.0 (2)	C4—C5—C6—C1	−0.3 (5)
O1—P1—O3—Ni1	−177.58 (19)	P1—O2—C8—C13	−91.2 (4)
O2—P1—O3—Ni1	73.3 (2)	P1—O2—C8—C9	91.8 (3)
S1—P1—O3—Ni1	−51.7 (2)	C13—C8—C9—C10	−1.4 (5)
N1—Ni1—O3—P1	−144.9 (2)	O2—C8—C9—C10	175.6 (3)
N1^i^—Ni1—O3—P1	35.1 (2)	C8—C9—C10—C11	1.2 (5)
N2—Ni1—O3—P1	−60.0 (2)	C9—C10—C11—C12	−0.3 (5)
N2^i^—Ni1—O3—P1	120.0 (2)	C9—C10—C11—C14	−179.8 (3)
N2—Ni1—N1—C20	−143.3 (2)	C10—C11—C12—C13	−0.6 (6)
N2^i^—Ni1—N1—C20	36.7 (2)	C14—C11—C12—C13	178.9 (3)
O3^i^—Ni1—N1—C20	123.1 (2)	C9—C8—C13—C12	0.6 (5)
O3—Ni1—N1—C20	−56.9 (2)	O2—C8—C13—C12	−176.3 (3)
N2—Ni1—N1—C19	−15.08 (19)	C11—C12—C13—C8	0.5 (5)
N2^i^—Ni1—N1—C19	164.92 (19)	C18—N2—C17—C21^i^	−173.6 (3)
O3^i^—Ni1—N1—C19	−108.67 (19)	Ni1—N2—C17—C21^i^	−41.9 (3)
O3—Ni1—N1—C19	71.33 (19)	C18—N2—C17—C16	−51.1 (4)
N1—Ni1—N2—C18	−15.2 (2)	Ni1—N2—C17—C16	80.6 (3)
N1^i^—Ni1—N2—C18	164.8 (2)	C18—N2—C17—C15	69.2 (4)
O3^i^—Ni1—N2—C18	75.0 (2)	Ni1—N2—C17—C15	−159.1 (2)
O3—Ni1—N2—C18	−105.0 (2)	C17—N2—C18—C19	−179.2 (3)
N1—Ni1—N2—C17	−150.8 (2)	Ni1—N2—C18—C19	42.2 (3)
N1^i^—Ni1—N2—C17	29.2 (2)	C20—N1—C19—C18	170.7 (3)
O3^i^—Ni1—N2—C17	−60.6 (2)	Ni1—N1—C19—C18	42.7 (3)
O3—Ni1—N2—C17	119.4 (2)	N2—C18—C19—N1	−58.6 (3)
P1—O1—C1—C2	−52.2 (4)	C19—N1—C20—C21	177.5 (3)
P1—O1—C1—C6	130.7 (3)	Ni1—N1—C20—C21	−59.3 (3)
C6—C1—C2—C3	0.7 (5)	C19—N1—C20—C22	55.2 (4)
O1—C1—C2—C3	−176.3 (3)	Ni1—N1—C20—C22	178.4 (2)
C1—C2—C3—C4	−0.3 (5)	N1—C20—C21—C17^i^	79.5 (4)
C2—C3—C4—C5	−0.4 (5)	C22—C20—C21—C17^i^	−157.2 (3)

Symmetry codes: (i) −*x*+1, −*y*+1, −*z*+1.

### Hydrogen-bond geometry (Å, °)

**Table:**

*D*—H···*A*	*D*—H	H···*A*	*D*···*A*	*D*—H···*A*
N1—H1···S1^i^	0.92 (3)	2.66 (3)	3.574 (3)	171 (3)
N2—H2···O2	0.96 (3)	2.27 (3)	3.234 (3)	178 (3)

Symmetry codes: (i) −*x*+1, −*y*+1, −*z*+1.
